# Post-vaccine rotavirus genotype distribution in Nairobi County, Kenya

**DOI:** 10.1016/j.ijid.2020.09.005

**Published:** 2020-11

**Authors:** Joshua Ndung’u Gikonyo, Betty Mbatia, Patrick W. Okanya, George F.O. Obiero, Carlene Sang, Duncan Steele, James Nyangao

**Affiliations:** a Department of Biochemistry and Biotechnology, The Technical University of Kenya (TU-K), PO Box 52428-00200, Nairobi, Kenya; b School of Pharmacy and Health Sciences, United States International University (USIU) – Africa, PO Box 14634-00800, Nairobi, Kenya; c Kenya Medical Research Institute (KEMRI), PO Box 43640-00100, Nairobi, Kenya; d Enteric and Diarrhoeal Diseases, Global Health Bill and Melinda Gates Foundation PO Box 23350, Seattle, WA98102, USA

**Keywords:** Rotarix, Rotavirus, Gastroenteritis, Genotypes, Vaccine

## Abstract

**Background:**

Rotaviruses are primary etiological agents of gastroenteritis in young children. In Kenya, G1P8 monovalent vaccine (Rotarix) was introduced in July 2014 for mandatory vaccination of all newborns at 6 and 10 weeks of age. Since then, no studies have been done to identify the rotavirus genotypes circulating in Nairobi County, Kenya, following the vaccine introduction, hence the post-vaccine genotype distribution is not known.

**Objectives:**

The aim of this study was to determine the post-vaccine occurrence of rotavirus genotypes in children <5 years of age in Nairobi County, Kenya.

**Methods:**

Stool samples were collected from children presenting with diarrhea for whom the vaccination status was card-confirmed. Fecal samples were analyzed for rotavirus antigen using a commercial enzyme immunoassay (EIA) kit, followed by characterization by polyacrylamide gel electrophoresis, RT-PCR, and nested PCR genotyping, targeting the most medically important genotypes.

**Results:**

The strains observed included G1P[8] (38.8%), G9P[8] (20.4%), G2P[4] (12.2%), G3[P4] (6.1%), G2P[6] (4.1%), and G9P[6] (4.1%). Mixed genotype constellations G3P[4][8] were also detected (4.1%). Remarkably, an increased prevalence of G2 genotypes was observed, revealing a change in genetic diversity of rotavirus strains. While the dominance of G1P[8] decreased after vaccination, an upsurge in G2P[4] (12.2%) and G9P[8] (20.4%) was observed. Additionally, G3[P4] (6.1%) and G2P[6] (4.1%) prevalence increased over the 3 years of study.

**Conclusions:**

The results inform the need for robust longitudinal surveillance and epidemiological studies to assess the long-term interaction between rotavirus vaccine and strain ecology.

## Highlights

• This article reports post-vaccine rotavirus G and P genotypes in Nairobi County.
• G1P[8] dominance has decreased following the introduction of Rotarix.
• There has been an increased prevalence of G2 genotypes following Rotarix introduction.
• There has been a change in genetic diversity of rotavirus strains in Nairobi, Kenya.

## Introduction

Globally, group A rotaviruses (RVA) are the primary causative agents of acute gastroenteritis requiring hospitalization among infants and young children <5 years of age (Banyai et al., 2012, Troeger et al., 2018). In 2016, RVA caused 128 500 deaths in children under 5 years of age globally (Fujii et al., 2019), of which 104 733 deaths occurred in sub-Saharan Africa (Damanka et al., 2019). The majority of these deaths occur in low-income countries, particularly those in sub-Saharan Africa, due to a lack of timely and appropriate treatment for dehydration (Wandera et al., 2017a). In Kenya, prior to the introduction of rotavirus vaccine, RVA caused more than 3908 infant deaths, 3015 outpatient visits, and 279 hospitalizations per 100 000 children <5 years of age annually, with an annual cost to the healthcare system of US$ 10.8 million (Wandera et al., 2017a).

Rotaviruses are members of the genus *Rotavirus* within the *Reoviridae* family. They are classified into eight groups (A–H) according to the antigenic and genetic features, and more recently by the amino acid sequences of the viral protein six (VP6) capsid protein, the sole component of the intermediate protein layer (Matthijnssens et al., 2010). The outer capsid layer of rotavirus is composed of two proteins encoded by the VP4 and VP7 genes (Thapar and Sanderson, 2004). VP4 defines P (protease sensitive) and VP7 defines G (glycoprotein), the serotypes of the virus. To date, 27 G-types and 35 P-types have been described in humans and animals worldwide (Matthijnssens et al., 2011, Martella et al., 2010). It is known that rotavirus strains belonging to different groups do not exchange their genome segments, i.e., reassort their genomes, during co-infection. In contrast, virus strains belonging to the same group can reassort their genomes, providing a mechanism for the evolution of novel genotypes (Aoki et al., 2009). Human rotavirus strains have been shown to carry 12 G and 15 P type specificities (Banyai et al., 2012). The surface proteins, VP7 and VP4, elicit neutralizing antibodies independently in vivo, therefore they are the main targets for vaccine development (Yuan et al., 2009).

The most common rotaviruses in humans belong to group A (RVA), accounting for nearly all rotavirus-associated mortality and morbidity. These RVA are targeted in the current vaccination programs. Other than RVA, groups B, C, and H rotaviruses are also implicated in gastroenteritis in humans; most notably group B, also known as adult diarrhea rotavirus (ADRV), which causes severe diarrhea in adults (Matthijnssens et al., 2011). Group C rotaviruses mainly infect children between 4 and 7 years of age, with each epidemic being sporadic and self-limiting in nature, while groups D, E, and G rotaviruses are only known to infect avian species (Tojnar et al., 2010).

Until 2009, there had been no effective rotavirus vaccine included in any national immunization program (Mpabalwani et al., 2016a). The World Health Organization (WHO) has since recommended two oral rotavirus vaccines for use in more than 100 countries worldwide: a two-dose human-attenuated rotavirus vaccine (Rotarix; GlaxoSmithKline Biologicals, Rixensart, Belgium) and a three-dose live pentavalent bovine–human reassortant vaccine (Rotateq; Merck, Whitehouse Station, NJ, USA) (WHO, 2009). Both vaccines have demonstrated an established efficacy against severe rotavirus infection (85–98%) and against acute gastroenteritis (AGE) (42–59%) in clinical trials (Vesikari et al., 2006; Ruiz-palacios et al., 2006). Impressive declines in hospitalizations and deaths resulting from AGE have been observed in many high and middle-income countries in the Americas and Europe, following the introduction of rotavirus vaccinations (Wandera et al., 2017a). In addition, declines in rotavirus disease among children who are not vaccine eligible have also been observed, a phenomenon referred to as herd immunity (Payne et al., 2011).

Genotypic studies in the pre-vaccine period in Nairobi County, Kenya, showed that G1 was the most prevalent among the G types, while P[8] was leading among the P types (Gikonyo et al., 2017). However, elsewhere, G3 was found to be the predominant genotype and emerged as a significant type with a detection rate of 40% among HIV-infected children with rotavirus infections in Nairobi, Kenya (Kiulia et al., 2009a). These studies indicate a notable relative shift in the prevalence of circulating rotavirus genotypes, and continuous monitoring and identification of their trend and prevalence is required, especially in the post-vaccine period.

Live-attenuated rotavirus vaccine (Rotarix) was introduced in July 2014 into the Kenyan Expanded Program on Immunization (EPI) through co-financing with the Global Alliance for Vaccine and Immunization (GAVI) Alliance. The vaccine is administered orally at 6 and 10 weeks of age and is aimed at protecting over 1.5 million children in the country from severe diarrhea (WHO, 2009). Thus, the onset of rotavirus vaccination in Kenya has provided a platform to assess the real-world impact of the vaccine in preventing and reducing the health burden of severe childhood diarrhea in the country.

Although Rotarix was highly efficacious for preventing severe rotavirus gastroenteritis in phase III trials in Latin America and Europe, it appears less effective in preventing diarrhea caused by G2P[4] RVA strains, which share neither the VP7 nor the VP4 surface antigen with the vaccine strains (Filipe et al., 2009). Since new rotavirus strains are being detected all over the world (Kiulia et al., 2014), rotavirus genotyping and continuous monitoring of the emerging strains remains critical in understanding the efficacy of the new rotavirus vaccines.

In July 2014, Kenya introduced Rotarix into its national immunization program for vaccination of all newborns at 6 and 10 weeks of age. No studies have yet been performed to identify the rotavirus genotypes circulating in Nairobi County following the introduction of the vaccine, and therefore the post-vaccine genotype distribution is not known. This hospital-based surveillance study in Nairobi County was therefore performed to provide molecular data for the genotype distribution. The study was performed between January 2015 and December 2017. Here, we report the rotavirus genotype distribution in Nairobi County, Kenya, 3 years following vaccine introduction.

## Materials and methods

### Study setup

The study was conducted in Nairobi County, Kenya. The Vaccines and Immunization Services Unit in the county launched the universal rotavirus vaccination program starting July 2014. A 3-year (2015–2017) cross-sectional study was designed to assess rotavirus strain distribution following vaccine introduction for the children of Nairobi County, Kenya. The recommendations in the WHO generic protocol for detecting strain distribution following vaccine introduction were followed.

The study focused on children under 5 years of age, targeting both outpatient and inpatient children presenting with severe AGE, experiencing episodes of three watery stools in a 24-h period for not more than 7 days, and with or without episodes of vomiting (WHO, 2015). The children either came directly from the community or were referred from peripheral community health centers and dispensaries. Decisions regarding hospitalization, investigations, and treatment were at the discretion of the attending clinicians, while vaccination status was card-confirmed.

### Sample collection and handling

Stool samples were collected from the children who met all of the inclusion criteria using a pathology investigation form adapted from the WHO generic protocol for rotavirus surveillance (WHO, 2015). Two hospitals in Nairobi County were chosen on the basis of WHO rotavirus surveillance guidelines recommending the selection of hospitals that admit >250 children for gastroenteritis per year. The selected hospitals belong to different geographical regions within the county: Mbagathi County Hospital in Kibra Sub County and Mama Lucy County Hospital in Embakasi West Sub County. The two county hospitals are major referral hospitals in Nairobi County; this ensured capturing most of the rotavirus infections in the region.

In this study, 323 fecal samples were collected in sterile stool caps and each sample was tagged with a date of collection and assigned a sample number. The samples were first stored at <4 °C in the hospitals and were later transported to the Rotavirus Laboratory at the Kenya Medical Research Institute (KEMRI), where they were stored at −20 °C awaiting processing.

### Target population

The study involved both inpatients and outpatients between January 2015 and December 2017. Focus was placed on children under 5 years of age, targeting the children who presented with severe AGE and had experienced an episode of three looser than normal or watery stools in a 24 -h period, for not more than 7 days, with or without episodes of vomiting. This is because rotavirus-based AGE symptoms generally resolve within 3–7 days (WHO, 2015). The children either came directly from the community or were referred from peripheral community health centers and dispensaries. Decisions regarding hospitalization, investigations, and treatment were at the discretion of the attending clinicians, while vaccination status was card-confirmed.

### Detection of group A rotavirus

The presence of RVA antigen in stool samples was determined using a commercial enzyme immunoassay (EIA) kit (ProSpecT Rotavirus Kit; Oxoid Limited, UK) according to the manufacturer’s instructions. A fecal suspension (10%) was prepared in a 1.5-ml Eppendorf tube by adding 100 μL stool sample to 1 mL sample diluent from the kit; this was mixed by vortexing for 1 min and then left on the bench to settle. One hundred microliters of the fecal suspension was added to separate microwells, as well as the negative and the positive controls (kit provided). Two drops (100 μL) of enzyme conjugate were added to the wells and incubated at 20–30 °C for 60 min.

The wells were washed and 100 μL of tetramethylbenzidine (TMB) substrate was added to each well and incubated in darkness at 20–30 °C for 10 min. Finally, 100 μL of sulfuric acid (stop solution) was added to each well. The results were first read visually and later within 10–15 min at a wavelength of 450 nm using a spectrophotometer. The cut-off value was calculated by adding 0.200 absorbance units to the negative control value, which was then used as the standard of reference.

### Determining the rotavirus electropherotypes

Polyacrylamide gel electrophoresis (PAGE) was used to determine the electropherotype variability of the rotavirus strains by displaying the migration patterns of the 11 segments of double-strand RNA (dsRNA). All of the positive fecal suspensions (*n* = 49) were analyzed and the long and short electropherotypes were distinguished. A standard electrophoresis was performed, as described by Steele and Alexander (1987). Rotavirus dsRNA was extracted and electrophoresis was performed in 10% polyacrylamide slab gels using the discontinuous buffer system (Kiulia et al., 2009b). Fixing and silver nitrate staining were followed by visualization over an illuminator.

### Rotavirus cDNA synthesis

dsRNA of RVA was extracted using the TRIzol extraction method (WHO, 2011) and RT-PCR was performed on the rotavirus dsRNA. The dsRNA was denatured by boiling at 94 °C for 5 min, followed by chilling on ice. Thereafter, the dsRNA was reverse-transcribed by incubating with reverse transcriptase and deoxynucleotides for 25–30 min at 42 °C. The resultant cDNA was then amplified by multiplex PCR.

### Genotyping of the amplified VP7 and VP4 genes by nested PCR

The nested PCR master mix was prepared by adding 10 mM dNTPs, 25 mM MgCl_2,_ 10× *Taq* buffer, *Taq* polymerase, dH_2_O, and standard primers for each of the VP7 genotypes (G_1_, G_2_, G_3_, G_4_, G_8_, G_9_, and G_12_) and primer End9 to a clean Eppendorf tube. Then the primers for each VP4 genotype (P_4_, P_6_, P_8_, P_9_, P_10_) were added with the Con_3._ The final volume was multiplied by the number of samples (*n* = 49). Forty microliters of the master mix were added to each tube containing the amplified VP7/VP4 cDNA, and this was run for 30 cycles in a thermocycler. The amplicons were then electrophoresed in a 1% agarose gel and viewed under UV light.

## Results

### Rotavirus electropherotypes

**Figure 1:**
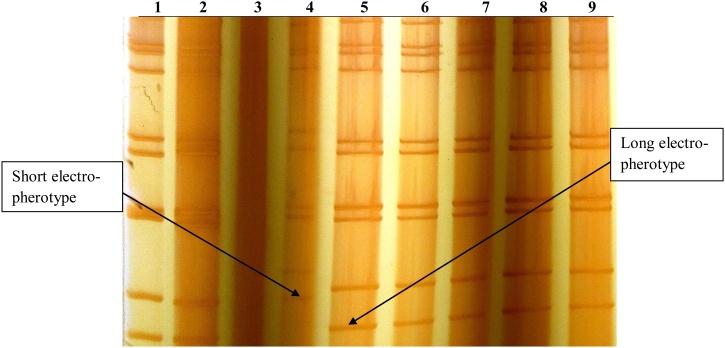
(1) PAGE migration patterns of the 11 rotavirus dsRNA segments.

A total of 323 samples were screened for RVA using ELISA techniques (155 from Mama Lucy County Hospital and 168 from Mbagathi County Hospital), of which 49 had a detectable rotavirus infection. Of the positive samples, 23 (46.9%) were from Mama Lucy County Hospital, while 26 (53.1%) were from Mbagathi County Hospital. Twenty-six (53%) were isolated from female children and 23 (47%) from male children. All rotavirus-positive fecal samples were analyzed by PAGE to characterize the rotavirus strains. Visible rotavirus dsRNA electrophoretic patterns were observed in 47 of the 49 ELISA-positive specimens subjected to sodium dodecyl sulfate (SDS)-PAGE (Figure 1). All of the isolates exhibited a profile of RVA based on the characteristic 4–2–3–2 distribution pattern of the genomic RNA segment. Rotaviruses whose RNA profiles had fast-migrating RNA segments were labeled ‘long’ electropherotypes (Figure 1, lanes 1, 2, 5, 6, 7, 8, and 9), while those with RNA profiles characterized by slower migrating segments were labeled ‘short’ electropherotypes (Figure 1, lane 4). One lane (lane 3) did not display any visible electrophoretic patterns. Of the isolated strains, 87.2% were long electropherotypes, while 12.8% were short electropherotypes. There were no profiles of mixed electropherotype infections detected.

The distribution of long RNA electropherotypes did not appear to vary with age and they occurred in all ages under study (≤5 years), while the short RNA profiles were identified among children aged up to 2 years old (Table 1).

**Table 1:** Distribution of rotavirus RNA electropherotypes among different age groups of children aged 5 years and below in Nairobi County.

Age group	Electropherotypes		
	Long patterns	Short patterns	Sub totals
0–6 months	4	0	4
7–12 months	6	2	8
13–18 months	8	3	11
19–24 months	12	0	12
25–36 months	9	1	10
37–60 months	2	0	2
Total	41	6	47

### The G and P genotypes detected

**Figure 2:**
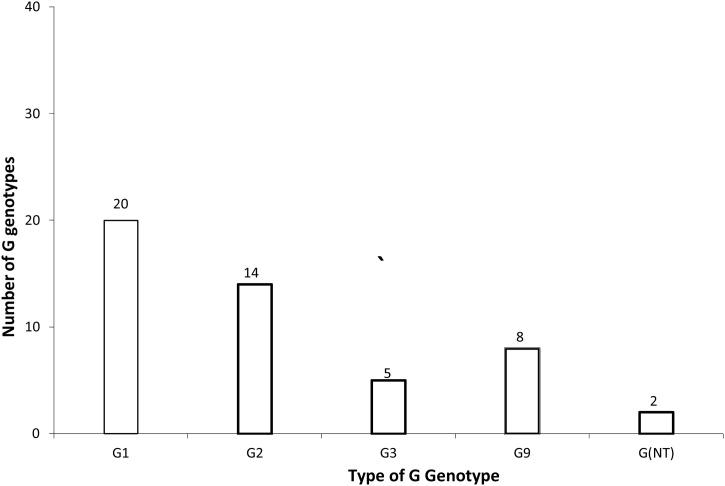
(2) Distribution of rotavirus G genotypes detected in Nairobi County between 2015 and 2017.

Four different G genotypes (G1, G2, G3, and G9) and three different P genotypes (P[4], P[6], and P[8]) were detected among the 49 positive samples. Among the G genotypes, G1 predominated (*n* = 20, 40.8%), followed by G9 (*n* = 14, 28.6%), G2 (*n* = 8, 16.3%), and G3 (*n* = 5, 10.2%). The G genotypes were not detected in the remaining two samples (G(NT)). In addition, mixed G genotypes were not detected in any of the samples (Figure 2).

**Figure 3:**
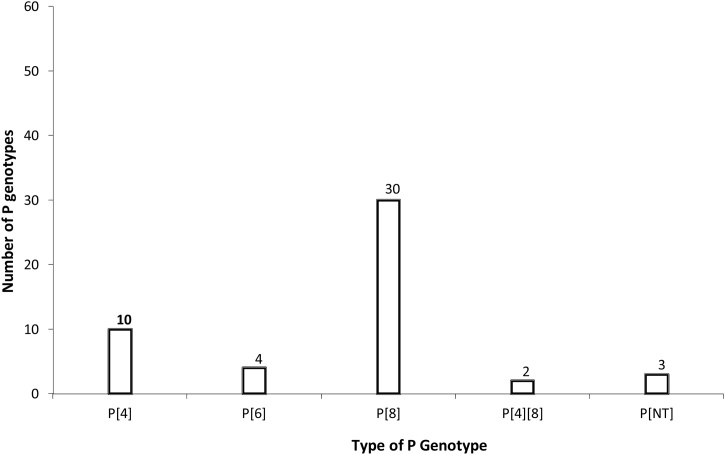
(3) Distribution of rotavirus P genotypes detected in Nairobi County between 2015 and 2017.

Of the P genotypes, P[8] predominated (*n* = 30, 61.2%), followed by P[4] (*n* = 10, 20.4%), P[6] (*n* = 4, 8.2%), and mixed infection P[4][8] (*n* = 2, 4.1%). In three samples (6.1%), the P genotype was not detected (P[NT]) (Figure 3).

### The combined distribution of G and P genotypes

The various G–P combinations observed in this study included G1P[8], G2P[4], G2[P6], G3P[4], G9P[6], G9P[8], and G3P[4][8]. G1P[8] was the most prevalent (38.8%), followed by G9P[8] (20.4%), G2P[4] (12.2%), G3[P4] (6.1%), G2P[6] (4.1%), and G9P[6] (4.1%). Furthermore, the single strain infections, combined mixed genotypes G3P[4][8] were detected in 4.1% of the cases. There were differences observed in the G–P genotype distribution in the years 2015, 2016, and 2017. G1P[8] was predominant in 2015 and 2017, while G9P[8] predominated in 2016 (Table 2).

**Table 2:** Post-vaccine yearly distribution of G–P genotypes of group A rotavirus between 2015 and 2017 in Nairobi County, Kenya.

G–P combination	Number of RVA strains in a year			
	2015	2016	2017	Total
G1P[8]	10	3	6	19
G2P[4]	3	5	2	10
G2P[6]	0	2	0	2
G3P[4]	0	2	1	3
G9P[6]	0	2	0	2
G9P[8]	2	3	1	6
Mixed	0	1	1	2
Partially untypeable	3	1	1	5
Sub-total	21	17	11	49

RVA, group A rotavirus.

## Discussion

Rotavirus vaccine has been in use in Kenya since July 2014. The WHO recommends that rotavirus vaccine be included in all national immunization programs, particularly those in high child mortality settings such as South Asia and sub-Saharan Africa, as part of a comprehensive package of diarrhea prevention and treatment measures (WHO, 2018). Rotavirus strains show genetic and antigenic diversity in terms of subgroup, electropherotypes, and the G and P genotypes (Vizzi et al., 2017). Following the introduction of a rotavirus vaccine, strain surveillance is of great importance in detecting the genotypic changes that may arise. At the molecular level, the genetic characteristics of rotaviruses can be followed by noting variation not only in rotavirus genotypes, but also in rotavirus electrophoretic patterns over a period of time (Tissera et al., 2017). The variability in strain prevalence in Nairobi County was assessed through the characterization of electrophoretic profiles and genotype analysis in this study. Hence, the study provides knowledge on rotavirus strains co-circulating in Nairobi County, 3 years after vaccine introduction.

PAGE of the rotavirus genome has been considered the most widely used technique for epidemiological studies involving the identification and characterization of rotaviruses (Mukhopadhya et al., 2017). We therefore employed PAGE in this study for effective characterization of the electrophoretic profiles. Of the 49 ELISA-positive samples that were subjected to SDS-PAGE, 47 displayed visible rotavirus dsRNA electrophoretic patterns (Figure 1).

Two RNA migration patterns or electropherotypes have been defined by SDS-PAGE for RVA: the ‘long’ type, defined by faster migration of the 10th and 11th segments, and the ‘short’ type, defined by slower migration of the 10th and 11th segments of the rotavirus RNA genome (Cláudia et al., 2005). Across the 3 years of this study, the two different electropherotypes co-circulated in Nairobi County, with the long strain being predominant and persistent. The long electropherotypes were always associated with G1 and G9 genotypes, whereas the short electropherotypes were associated with genotype G2. However, it was noted that the pattern of the electropherotype suggested but did not confirm a particular genotype, as described by Sethi et al. (2006). Generally, according to Claudia et al. (2005), the predominance of rotavirus strains with the long electropherotype correlates with the most common Wa genogroup, which comprises most rotavirus strains of serotypes G1, G3, G4 and G9, whereas the rotavirus strains displaying the short electropherotype are usually included in the less prevalent DS-1 genogroup, which is composed mainly of the G2 strains. This has further been confirmed by research done in Turkey, where all G9P[8] strains identified in a study on the diversity of human rotavirus G9 were of the long electropherotype except one that exhibited a short electropherotype (Gulendam et al., 2008).

Reassortment of the VP7 and VP4 genes into both ‘long’ and ‘short’ electropherotype strains has resulted in a great variety of genomic constellations exhibited in different countries (Banyai et al., 2012). For example, G9P[4] ‘short’ electropherotype strains have been identified in Thailand, while G9P[4] ‘long’ electropherotype strains have been found in Brazil (Estes and Kapikian, 2007). G9P[6] ‘short’ electropherotype strains have been detected in the USA and Bangladesh, while the ‘long’ electropherotype strains of the same G9P[6] have been detected in India. G9P[8] ‘long’ electropherotype strains have been shown to circulate in Libya, Cuba, and Japan (Grassly and Fraser, 2006, Iturriza et al., 2002). Hence, the divergence of the Nairobi strains into short and long electropherotypes of the genotypes observed in this study (G1P[8], G2P[4], G2[P6], G3P[4], G9P[6], and G9P[8]) suggests that all of the strains were not evolved locally from one progeny but from different progenies.

Previous studies in Nairobi County in the pre-vaccine period showed remarkable global frequencies of co-circulating G genotypes G1, G3, and G9, and P genotypes P[4], P[6], and P[8] (Gikonyo et al., 2017). In the current study, we found some diversity of rotavirus strains including four different G genotypes and three different P genotypes, previously identified. Although strain G1P[8] still predominated after vaccine introduction, it was at a reduced prevalence compared to the pre-vaccine period (Wandera et al., 2017a). Worth noting, an upsurge in the strains fully and partially heterotypic to the vaccine strain, i.e., G9P[8] (20.4%), G2P[4] (12.2%), G3[P4] (6.1%), and G2P[6] (4.1%), was observed relative to the pre-vaccine period. Recently, a similar increase in G2P[4] (17%) prevalence was reported among Kenyan peri-urban children in the post-vaccine period, in addition to G3P[8] (13%) and G3P[6] (7%) (Wandera et al., 2017) that were not detected in the present study. These indicate both mixed and temporal rotavirus genotype prevalence in Nairobi post-vaccination.

In Belgium, where Rotarix is the main vaccine, a substantial long-term increase in the proportion of G2P[4] strain genotype was noted after vaccine introduction among children (Pitzer et al., 2015). This suggests that Rotarix exerts selective pressure on the distribution of RVA genotypes, leading to the observed fluctuations in prevalence. Although changes in the distribution of the RVA genotype following mass vaccinations with Rotateq and/or Rotarix have been observed in Brazil, the USA, and some Australian states (Mpabalwani et al., 2016b), it is not presently clear that these changes can be solely attributed to the vaccines. In Malawi, the G2 genotype was found to dominate in the season following Rotarix introduction, and a follow-up study attributed the increase in G2 incidence to temporal oscillations (Bar-Zeev et al., 2016). This indicates that non-vaccine target genotypes fluctuate in their prevalence in different regions.

Previously in Kenya, similar results of temporal fluctuations in RVA genotypes were reported in the pre-vaccine period (Nyangao et al., 2010, Nokes et al., 2010). Comparatively, fluctuations in the G–P genotype distribution were observed year by year before and after vaccine introduction in Kenya (Wandera et al., 2017a, Wandera et al., 2017b). Given that the fluctuations emerged in both the pre- and post-vaccine periods, the genotypic changes cannot be directly linked to the effects of the vaccine per se.

### Limitations

Due to the limitations of this study, we could not attribute the changing prevalence of genotypes to vaccine-induced selective pressure. The limitations include the absence of data on the strain-specific effectiveness of vaccine and the limited post-vaccine observation period, which did not permit monitoring for any sustained predominance of the genotypes. In addition, challenges with record-keeping in the hospitals did not permit the evaluation of rotavirus vaccination status of each individual child, thus making it difficult to make a comparison between the vaccinated and unvaccinated children.

### Conclusions

Following the introduction of the rotavirus vaccine in 2014, the rotavirus genotype distribution and diversity has changed in Kenya, with a large relative shift towards the non-G1P[8] strains. These changes will need continued surveillance, especially as the number and age of the vaccinated birth cohort increase over the coming years. The results of this study and continued genotype surveillance across Kenya in the coming years will help to inform whether future modifications to rotavirus vaccines may be needed.

## Author contributions

Joshua Gikonyo: developed the study protocol, carried out sample collection, handled the laboratory work and drafted the manuscript. Betty Mbatia: oversaw the overall scientific integrity of the study, supervised sample and data collection and project implementation, drafted and edited the final manuscript. Patrick W. Okanya: supervised data collection, edited the project report, contributed to data analysis and edited the final manuscript. George F.O. Obiero: contributed to data analysis and edited the final manuscript. Carlene Sang: carried out the laboratory work. Duncan Steele: final edited the manuscript. James Nyangao: developed the study protocol, carried out the laboratory work and oversaw project implementation. All authors read and approved the final manuscript.

## Funding

This work was supported by; 1. The Kenya National Research Fund (NRF), [grant number NRF/2334/2017], and, 2. Alexander von Humboldt Foundation Return Home Fellowship through Dr. George F.O. Obiero during the manuscript preparation [grant number KEN_11848_GF-P].

## Ethical considerations

Ethical clearance was sought from the Kenya Medical Research Institute Scientific and Ethics Review Unit (SSC No. 015/3368). Informed written consent was sought from the parents or guardians of all the recruited children. All personal identification was removed from the fecal samples sent to the laboratory for viral gastroenteritis analysis and new laboratory numbers were assigned.

## Conflict of interest

The authors declare that they have no known competing financial interests or personal relationships that could have appeared to influence the work reported in this paper.
